# Optimising Intraoperative Fluid Management in Patients Treated with Adolescent Idiopathic Scoliosis—A Novel Strategy for Improving Outcomes

**DOI:** 10.3390/children10081371

**Published:** 2023-08-10

**Authors:** Jakub Miegoń, Sławomir Zacha, Karolina Skonieczna-Żydecka, Agata Wiczk-Bratkowska, Agata Andrzejewska, Konrad Jarosz, Monika Deptuła-Jarosz, Jowita Biernawska

**Affiliations:** 1 Department of Anaesthesiology and Intensive Care, Pomeranian Medical University in Szczecin, 71-252 Szczecin, Poland; 2 Department of Paediatric Orthopaedics and Oncology of the Musculoskeletal System, Pomeranian Medical University in Szczecin, 71-252 Szczecin, Poland; 3 Department of Biochemical Science, Pomeranian Medical University in Szczecin, 71-460 Szczecin, Poland; 4 Department of Clinical Nursing, Pomeranian Medical University in Szczecin, 71-210 Szczecin, Poland; 5 Department of Neurosurgery and Paediatric Neurosurgery, Pomeranian Medical University in Szczecin, 71-252 Szczecin, Poland

**Keywords:** adolescent idiopathic scoliosis, haemodynamic monitoring, hypotension, length of stay, ERAS (enhanced recovery after surgery)

## Abstract

Scoliosis surgery is a challenge for the entire team in terms of safety, and its accomplishment requires the utilization of advanced monitoring technologies. A prospective, single centre, non-randomised controlled cohort study, was designed to assess the efficacy of protocolised intraoperative haemodynamic monitoring and goal-directed therapy in relation to patient outcomes following posterior fusion surgery for adolescent idiopathic scoliosis (AIS). The control group (*n* = 35, mean age: 15 years) received standard blood pressure management during the surgical procedure, whereas the intervention group (*n* = 35, mean age: 14 years) underwent minimally invasive haemodynamic monitoring. Arterial pulse contour analysis (APCO) devices were employed, along with goal-directed therapy protocol centered on achieving target mean arterial pressure and stroke volume. This was facilitated through the application of crystalloid boluses, ephedrine, and noradrenaline. The intervention group was subjected to a comprehensive protocol following Enhanced Recovery After Surgery (ERAS) principles. Remarkably, the intervention group exhibited notable advantages (*p* < 0.05), including reduced hospital stay durations (median 7 days vs. 10), shorter episodes of hypotension (mean arterial pressure < 60 mmHg—median 8 vs. 40 min), lesser declines in postoperative haemoglobin levels (−2.36 g/dl vs. −3.83 g/dl), and quicker extubation times. These compelling findings strongly imply that the integration of targeted interventions during the intraoperative care of AIS patients undergoing posterior fusion enhance a set of treatment outcomes.

## 1. Introduction

Posterior fusion surgery for adolescent idiopathic scoliosis (AIS) poses a significant challenge to anaesthetists due to the extensive surgical area and the need for specific anaesthesia techniques that accommodate intraoperative neurophysiological monitoring of the spinal cord. AIS, the most common spinal deformity in children, necessitates surgical correction while minimising the risk of spinal cord injury, which can be achieved through intraoperative neuromonitoring and the maintenance of adequate spinal cord perfusion. In our centre, when qualifying for scoliosis, the criteria outlined in the Scoliosis Research Society (SRS) guidelines are followed, which means that scoliosis surgery is indicated for patients above 12 years of age with a Cobb angle > 45 degrees, without the necessity of general symptoms. For patients with early-onset scoliosis, two criteria must be met-both a Cobb angle > 45 degrees and a progression of more than 10 degrees per year [1]. The orthopaedics do not wait for complications in the form of organ dysfunction to arise. If the Cobb angle is less than 45 degrees, conservative treatment of scoliosis is possible. For angles below 30 degrees, rehabilitation is applied, and for angles above 30 degrees, bracing is considered, provided that the patient cooperates with this therapy.The maintenance of intraoperative haemodynamic stability holds significant importance in determining the postoperative prognosis of AIS patients undergoing surgical intervention. Hypotension, leading to tissue hypoperfusion, is a key contributor to unfavourable neurological outcomes. Monitoring parameters such as intraoperative volemia, cardiac function, blood pressure, and haemoglobin levels play a pivotal role in ensuring sufficient oxygen delivery to vital organs. Tissue hypoxia stands out as a major driver of perioperative complications [2]. Appropriate management of fluid administration and vasopressors becomes essential in preventing and treating hypo- and hypervolemia, while maintaining adequate oxygen delivery without causing fluid overload.

Intraoperative hypotension (IOH) frequently occurs during surgical procedures. However, a universally accepted definition for IOH remains elusive. IOH poses the potential for ischemia-reperfusion injury, which can manifest as dysfunction in vital organs [3]. While some studies have reported the association of IOH with postoperative complications in adults, similar investigations in children are scarce. The APRICOT study documented hypotension in 54.9% of major cardiovascular events, with the majority of these events (94%) resulting in uneventful outcomes [4].

Standard methods based on non-invasive blood pressure monitoring may not be sufficient to detect perfusion abnormalities. Hypovolemia can mask itself with normal blood pressure due to increased vascular resistance, and anaesthesia can influence heart rate, making it an unreliable indicator of hypovolemia. Moreover, oscillometric techniques, in comparison to invasive blood pressure measurement, tend to overestimate low blood pressure readings and underestimate high blood pressure readings. Consequently, more advanced haemodynamic monitoring methods have been developed.

Uncalibrated devices utilise patient anthropometric and demographic data, along with internal databases and algorithms, to calculate cardiac output (CO) from arterial waveforms, which proves valuable in perioperative optimisation protocols. Measured, calculated or derived haemodynamic parameters are instrumental in identifying the underlying causes of hypotension.

Goal-directed therapy (GDT) constitutes a haemodynamic treatment approach that involves titrating fluid and inotropic agents based on physiological flow-related endpoints [5]. Its implementation has shown to reduce perioperative morbidity and mortality in both adult and paediatric populations [6,7,8,9,10,11,12,13]. However, the application of monitoring methods primarily developed for adults to children may introduce challenges due to differing characteristics of the vascular system and the absence of reference values [14,15].

Paediatric patients exhibit a poor correlation between advanced haemodynamic parameters routinely used in adults, such as arterial pressure or plethysmographically derived variables, and fluid responsiveness [14]. This discrepancy in correlation with adults can be attributed to greater vascular compliance in paediatric patients. However, the precise age at which arterial properties in children resemble those in adults remains unknown, necessitating further research.

The objective of this study was to assess the effectiveness of a protocolised care approach involving intraoperative haemodynamic monitoring and goal-directed therapy in improving patient outcomes following posterior fusion surgery for AIS.

## 2. Materials and Methods

A prospective, single-centre, non-randomised, controlled cohort study was employed. The research focused on 70 consecutive Caucasian teenagers with AIS who underwent posterior fusion. The criteria for inclusion in the study were as follows: individuals who had their first scoliosis operation and were under 18 years of age. Patients who met the qualification for scoliosis surgery at this facility underwent X-ray imaging, computed tomography, and magnetic resonance imaging. Those with a Cobb angle exceeding 45 degrees were deemed eligible. Prior to anaesthesia approval, echocardiography and spirometry tests were conducted. Criteria used to exclude patients from the study included emergency surgery, reoperation, advanced chronic respiratory-circulatory failure, or scoliosis caused by factors other than idiopathic origins.

**Figure 1:**
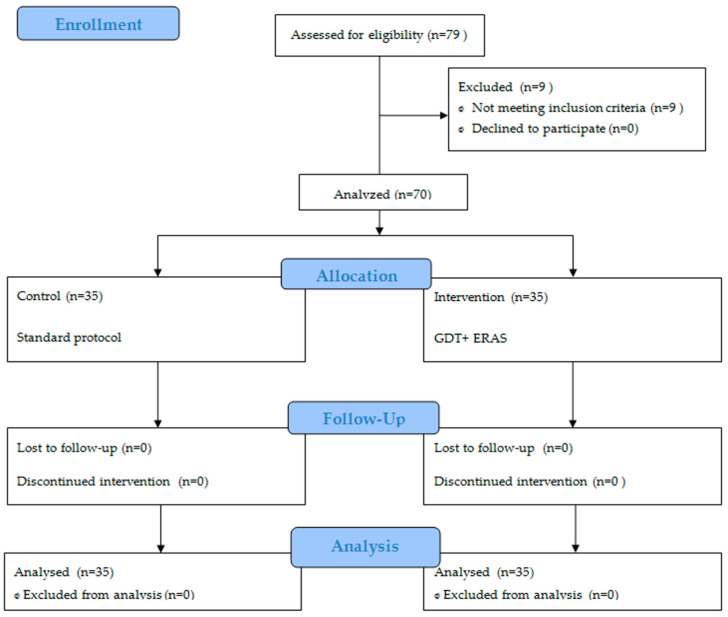
The Consort diagram.

The control group, comprising of 35 patients with a mean age of 15 years, including 7 boys, received standard blood pressure management during the surgical procedure. Subsequently, the obtained results were analyzed to establish the protocol for the intervention group. The intervention group consisted of 35 patients, with a mean age of 14 years and a higher proportion of females (5 boys). This group followed a protocol that involved intraoperative minimally invasive haemodynamic monitoring, GDT, and components of Enhanced Recovery After Surgery (ERAS). The study’s participant flow is visually represented in Figure 1 using the Consort diagram.

The study protocol was officially registered with the clinicaltrials.gov database and can be identified by the accession number NCT 05159505.

### 2.1. Preparation for Surgery

In preparation for the scheduled surgery, patients were directed to undergo an anaesthetic consultation and subsequently were qualified for general anaesthesia.

On the day of the operation, as a pre-emptive analgesia, patients were administered metamizole at a dosage of 15 mg/kg for children weighing under 50 kg, or a 1 g oral dose if body weight exceeded 50 kg. Additionally, they received an orally administered carbohydrate-rich fluid (Preop, Nutricia, Poland).

### 2.2. Surgery

**Figure 2:**
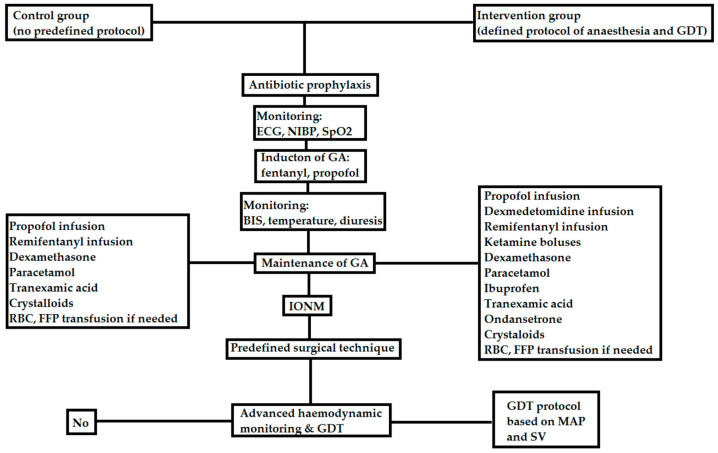
The anaesthesia protocol used in this study (ECG—electrocardiography, NIBP—non-invasive blood pressure, SpO2—pulse oximetry, GA—general anaesthesia, RBC—red blood cells, FFP—fresh frozen plasma, IONM—intraoperative neuromonitoring, GDT—goal directed therapy, MAP—mean arterial pressure, SV—stroke volume).

During the procedure, all patients received general anaesthesia, which involved intubation using a reinforced endotracheal tube and mechanical ventilation using a Primus apparatus (Dräger, Germany). The control group did not adhere to a specific anaesthesia protocol, as the decision regarding analgesic medications and anaesthesia management was left to the administering anaesthesiologist. A visual representation of the anaesthesia administration in the intervention group can be found in Figure 2. In this group, patients had a radial artery catheter placed for arterial access, and an HemoSphere (Edwards Lifesciences, Irvine, CA, USA) monitor and a Flotrac or Acumen IQ sensor was utilised for haemodynamic monitoring purposes.

**Figure 3:**
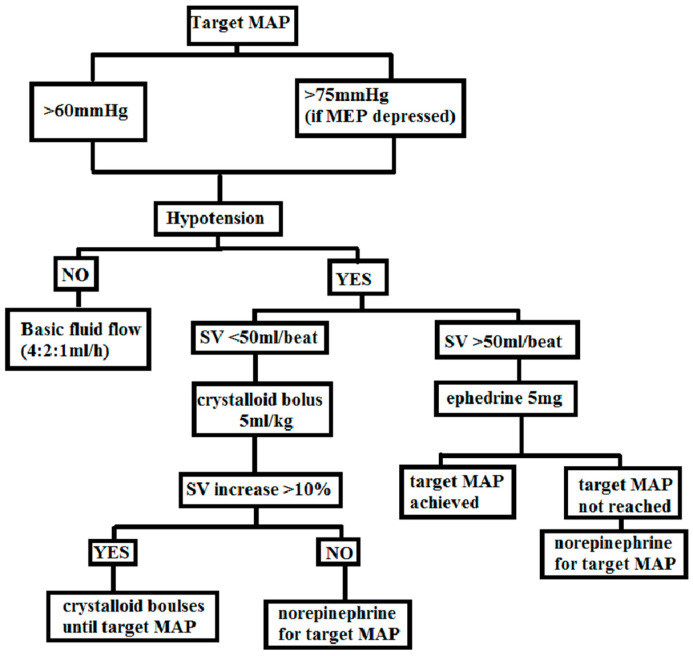
The GDT protocol used in this study, adapted from Edwards Lifesciences protocol [16] (MAP—mean arterial pressure, MEP—motor-evoked potentials, SV—stroke volume).

In this study, a GDT protocol was implemented, aiming to maintain the desired levels of mean arterial pressure (MAP) and stroke volume (SV) by means of fluid therapy and the administration of vasopressors. A detailed outline of the protocol can be found in Figure 3.

Fluid therapy in this study encompassed the administration of balanced crystalloid solutions (Sterofundin-B. Braun/Optilyte-Fresenius Kabi) intravenously at a rate of 4:2:1 mL/kg/h. The initiation of the protocol occurred when the patient was transitioned to the prone position, preceded by an intravenous bolus of 5 mg ephedrine to address hypotension. The target values aimed to maintain MAP above 60 mmHg, with a higher threshold of MAP > 75 mmHg in cases of depressed MEP.

If hypotension (MAP < 60 mmHg) and SV below 50 mL/beat were observed, a crystalloid bolus of 5 mL/kg was administered intravenously over a period of 10–15 min. Following the bolus, the response to fluid therapy was evaluated. If SV increased by more than 10% and hypotension persisted, an additional fluid bolus was given. In cases where SV did not increase by more than 10%, norepinephrine infusion was initiated to sustain a MAP above 60 mmHg.

When hypotension (MAP < 60 mmHg) was present but SV exceeded 50 ml/beat, a single dose of 5 mg ephedrine was administered. If the response was insufficient, norepinephrine infusion was initiated to maintain the target MAP. In situations where SV was below 50 mL/beat but no evidence of hypotension (MAP > 60 mmHg) or peripheral perfusion failure (capillary refill time < 2 s) was observed, no fluid bolus was administered. The use of colloids for fluid administration was prohibited. In cases where blood loss surpassed 7 mL/kg, a transfusion of 1 unit of red blood cells was provided.

The extubation criteria were not firmly defined in the study. They considered the presence of spontaneous breathing, the level of consciousness-responsiveness to voice with eye opening, the presence of gag reflex, and hemodynamic stability.

### 2.3. Surgical Technique

The surgical procedure employed a posterior approach, involving ligament and bone release, implant fixations, and the ultimate correction using titanium rods. The surgical techniques utilised either a screws-only system or hybrid systems incorporating screws, hooks, or sublaminar bands.

### 2.4. Intraoperative Neuromonitoring (IONM)

Continuous monitoring of motor and sensory potentials was carried out throughout the procedure to ensure the preservation of spinal cord function. The IONM was performed using the Inomed Neurstimulator ISIS device, following a consistent protocol. The placement of screws relative to the spinal root was verified using direct nerve stimulation (DNS) electromyography. DNS was conducted with a constant current (CC) of 3 Hz frequency, 200 µs pulse duration, monopolar/negative stimulation, and a stimulation threshold of 8 mA. Additionally, the integrity of the corticospinal pathway was assessed during the correction procedure using transcranial electrostimulation (TES) or motor evoked potentials (MEP). TES/MEP involved CC with 2 Hz frequency, interstimulus interval (ISI) of 4 mA, a train of 5 pulses, positive polarity, 500 µs pulse duration, and a maximum amplitude of 200 mA. The motor responses were recorded from indicator muscles of the lower extremities and upper extremity flexors, serving as a reference. Furthermore, somatosensory evoked potentials (SEP) were evaluated by stimulating the posterior tibial nerves with CC, square pulses/positive polarity, 200 µs pulse duration, 3.7 Hz frequency, and an amplitude of 25 mA, as an assessment of sensory pathway integrity.

### 2.5. Postoperative Course

Patients were monitored in the post-anaesthesia care unit (PACU) for a duration of 24 h after surgery. Vital signs were regularly assessed, and a numerical scale (NRS) was utilised to evaluate pain intensity. This assessment was performed every hour for the initial 24 h and then every 8 h until hospital discharge. The evaluation included monitoring the administered drugs, their type, dose, route, and any occurrence of side effects or complications.

During the postoperative period, medications such as paracetamol, metamizole, ibuprofen, magnesium sulfate, intravenous opioid infusion, and lide infusions were administered at regular intervals. The decision to transition from intravenous to oral treatment was determined based on the patient’s daily requirements.

The study maintained consistency in preoperative preparation, surgical technique, perioperative analgesia, and postoperative evaluation parameters between the “control” and “intervention” groups. The intervention group followed a hospitalisation plan rooted in the principles of the ERAS protocol. ERAS programs employ a multidisciplinary approach to improve surgical outcomes by implementing evidence-based, procedure-specific care protocols. In this study, the ERAS program encompassed elements such as early mobilisation, rehabilitation, early evacuation of drains, early initiation of oral hydration, and early initiation of feeding.

### 2.6. Definitions of Complications

The evaluation of the obtained results was a comparison in terms of: demographic data, total time of hypotension, duration of the surgical procedure “skin to skin” (minutes), duration of hospitalisation (days).

Hypotension in the study was defined as systolic blood pressure (SBP) below 90 mmHg and MAP below 60 mmHg for at least 1 min. Adverse drug reactions encompassed symptoms such as apnoea, dyspnoea, decrease in SpO2 below 90%, bradycardia, hypotension, pruritus, nausea, vomiting, urinary retention, constipation, dizziness and drowsiness preventing rehabilitation. Surgical complications included partial or complete spinal cord injury leading to transient or persistent paralysis, transient neuropraxia related to positioning, dural tear, position-related complications, visual disturbances, respiratory-circulatory failure, surgical site infection, haematoma, gastric disorders, pneumonia, and death. In order to assess the findings, a comparative analysis was conducted, which involved examining demographic data, total duration of hypotension, the length of the surgical procedure from “skin to skin” in minutes, and the duration of hospitalisation in days.

### 2.7. Outcomes

The primary outcome of the study was the duration of hospitalisation in days. Additionally, secondary outcomes were evaluated, including the following: intraoperative hypotension time (min), volume of red blood cell transfusion (mL), volume of fresh frozen plasma transfusion (mL), intraoperative blood loss (mL), administration of crystalloids (mL/kg), total dose of ephedrine (mg), pre-surgery haemoglobin level (g/dL), post-surgery haemoglobin level (g/dL), change in haemoglobin level (g/dL), pre-surgery haematocrit level (%), post-surgery haematocrit level (%), change in haematocrit level (%),the duration of the surgical procedure “skin to skin” (min), and time to extubation (min), the occurrence of neurological and cardiac complications.

### 2.8. Statistical Analyses

To assess the distribution of continuous variables, the Shapiro-Wilk normality test was conducted. Descriptive statistics were then reported using means and standard deviations, or median and interquartile ranges (IQR), as appropriate. Qualitative variables were presented as numbers and percentages. For comparing outcomes between the two groups, either the nonparametric Mann-Whitney test or parametric *t*-test was employed. The chi-squared test was utilised to analyze the association between qualitative variables. For the variables with low numbers (*n* < 5), a Fisher’s exact test was adopted. The significance level for type I error was set at 0.05, and the calculations were performed using MedCalc statistical software version 20.110 (Ostend, Belgium). To control for type I errors, the false discovery rate (FDR) approach was applied using the p.adjust function from the stats package in R [17]. The statistical power was determined using G*Power software version 3.1.9.2 [18].

## 3. Results

### 3.1. Study Participants

Table 1 presents the baseline characteristics of the study participants in relation to the tested procedure. No statistically significant differences were observed among any of the tested parameters except of haemoglobin level. Patients within the control group had a higher concentration as compared to intervention group counterparts.

### 3.2. Outcomes

Table 2 presents a comprehensive comparison of perioperative data, hypotension time, complications, transfusions, and haemoglobin levels between the control and intervention groups. The results demonstrate that intraoperative haemodynamic monitoring had a significant impact on various outcomes, including duration of hospital stay, number of neurological and cardiac complications, hypotension time, fresh frozen plasma (FFP) transfusion, time to extubation, and all tested blood parameters. Notably, the intervention group exhibited minimal duration of hypotension with MAP below 55 mmHg, with a median of 0 min and an interquartile range (IQR) of 0–3.5 min. The intervention group did not require the administration of norepinephrine throughout the study.

**Figure 4:**
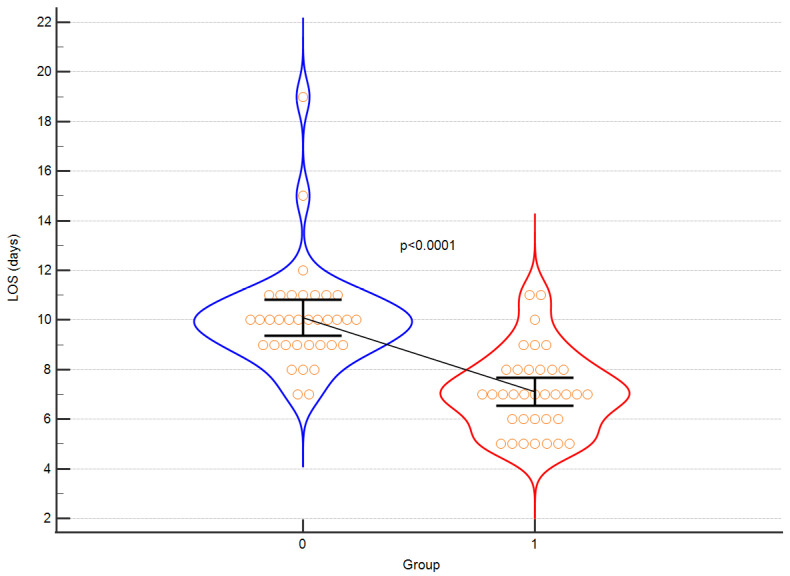
A violin plot depicting the length of hospital stay between control (0) and intervention (1) group. Orange circles represent individual cases. The black horizontal line connects means. Error bars represent SD.

**Figure 5:**
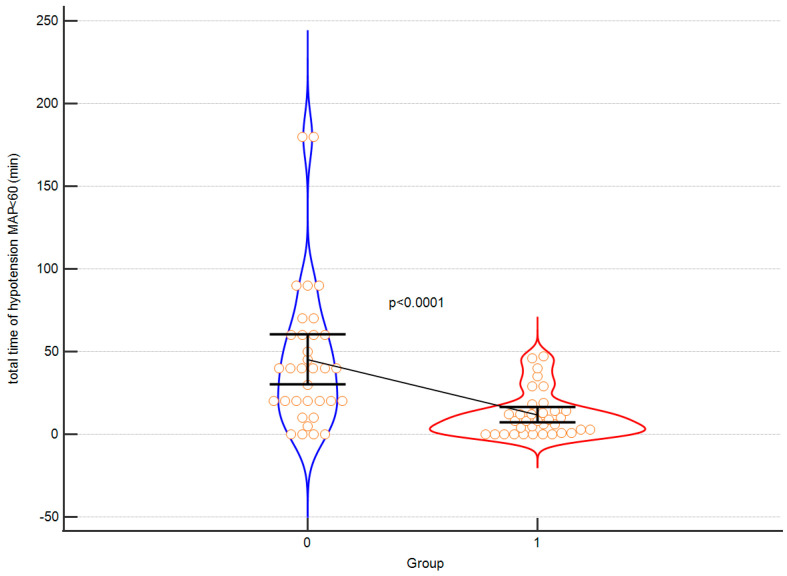
A violin plot depicting the perioperative hypotension time between control (0) and intervention (1) group. Orange circles represent individual cases. The black horizontal line connects medians. Error bars represent IQRs.

Exemplary Figure 4 and Figure 5 of significant results are presented below.

In the control group, exclusively, four complications of neurological and cardiac nature were observed (cardiorespiratory failure resulting from hypovolemic shock—three patients, transient limb paresis—one patient). None of these events occurred among the subjects in the intervention group. It is noteworthy to mention that the hypovolemic shock in the control group was due to a gradual rather than sudden blood loss, which eventually proved to be significant and life-threatening. Consequently, these patients required postoperative intensive care, and one of them additionally developed a complication in the form of pneumonia. Unfortunately, we were not able to assess by means of statistics whether the complications occur less frequently when an invasive haemodynamic monitoring is introduced, as no such data were obtained in the intervention group. More studies to verify these results are warranted.

## 4. Discussion

This study aimed to assess the efficacy of intraoperative haemodynamic monitoring GDT outcomes in adolescents undergoing posterior fusion for AIS.

Firstly, the intervention group demonstrated a significantly shorter length of stay in hospital. Furthermore, patients in the intervention group displayed a substantially reduced duration of hypotension (MAP < 60 mmHg). Additionally, it is worth mentioning that the intraoperative diuresis was higher in the control group, while the GDT approach resulted in more stable haemoglobin and haematocrit levels with a smaller amplitude of changes and improved blood pressure stability. The extent of blood loss remained similar in both groups, albeit FFP was more frequently transfused in the control group. Additionally, the time interval from the conclusion of surgery to extubation was notably shorter in the intervention group, which contributed to earlier patient mobilisation. The duration of surgical procedures did not exhibit any notable differences between the two groups, which can be attributed to the consistent involvement of the same surgical team. Remarkably, the control group experienced instances of cardiac and neurological complications, whereas no such adverse events were observed in the intervention group following the implementation of the GDT protocol. These findings emphasise the potential of the intervention to enhance post-operative recovery and overall patient outcomes. The results obtained by the authors were corrected for multiple testing and statistical power was calculated for each comparison to demonstrate the validity. As elegantly summarised in Table 2, the following outcomes gained high statistical power: duration of hospitalisation, hypotension time, FFP transfusion volume, change in haemoglobin, time to extubation. Also, in regard to these results, the differences between intervention group and control group remained statistically significant after multiple testing. The authors hereby confirm, that invasive haemodynamic monitoring in teenagers with AIS is efficient with respect to many of intra and postoperative parameters

The observed reduction in length of stay within our study can be attributed to a range of factors, with particular emphasis on the absence of complications within the intervention group and the successful implementation of the ERAS strategy for uncomplicated patients. A study conducted by Marsollier et al., which harnessed the ERAS protocol for AIS cases, demonstrated a noteworthy decrease in the median length of hospital stay within the ERAS group [19]. Additionally, research by Jeandel et al., focusing on ERAS utilization for AIS, showcased not only a decrease in hospital stay but also a significant reduction in hospitalization costs [20]. These findings collectively underscore the significance of the ERAS approach in optimizing postoperative outcomes and resource utilization in AIS cases.

According to surveys conducted by the Scoliosis Research Society, spinal deformity surgery carries a risk of spinal cord injury, with reported rates ranging from 0.3% to 0.6% [21]. These injuries to the spinal cord can arise from various factors, including direct compression by surgical instruments or implants, compromised blood flow due to vessel stretching or compression, interruption of radicular blood flow, spinal cord distraction injury, or the presence of epidural haematoma. Among these factors, ischemic injury is the most commonly observed, with the motor pathways supplied by the anterior spinal artery being particularly susceptible to such damage. Therefore, timely identification and prevention of hypotension during spinal deformity surgery are vital in reducing the risk of ischemic injury. However, detecting hypotension early can pose challenges, especially in paediatric patients, due to the limited availability of non-invasive monitoring options.

To address the aforementioned challenges, the utilisation of invasive monitoring and GDT emerges as a potential solution, given its proven advantages in adult populations. Hence, the objective of this study is to assess the potential advantages of GDT in minimizing the duration of hypotension episodes during scoliosis surgery, specifically focusing on the prevention of intraoperative spinal cord ischemia, which is recognised as the most prevalent complication.

The selection of a MAP threshold of less than 60 mmHg as an indicator of hypotension in this study was driven by several considerations. Notably, there are no established hypotension thresholds specific to the age group of teenagers undergoing spinal deformity surgery. Given that the study population consisted of slim teenagers who likely possessed good microcirculation autoregulation, the authors believed that a lower threshold would be appropriate for detecting deviations from normal blood pressure ranges. Additionally, previous research has demonstrated that maintaining a MAP above 60 mm Hg during spinal surgery is a critical factor in lowering the likelihood of spinal cord injury, aligning with the observations made by the authors [22]. This evidence supports the notion that maintaining an adequate perfusion pressure is crucial for safeguarding the integrity of the spinal cord. By selecting a MAP threshold of less than 60 mmHg, the authors aimed to establish a clinically relevant cut-off that would prompt timely intervention to prevent hypotension-related complications. This threshold takes into account the unique characteristics of the study population while aligning with the existing literature on the importance of maintaining appropriate blood pressure levels during spinal surgery. While the Flotrac APCO system has demonstrated utility in the paediatric population, it is important to note that its validation specifically in children is yet to be established.

The findings of this study demonstrate that the combination of invasive arterial pressure monitoring and the implementation of the GDT protocol leads to a significant reduction in the duration of intraoperative hypotension. Notably, it was observed that the instances where the MAP remained below 55 mmHg were remarkably transient, indicating effective management of blood pressure during the surgical procedure.

GDT protocols have gained significant popularity in the operating theatre as a means to optimise the patient’s haemodynamic status and enhance overall outcomes. Various types of GDT protocols can be employed, such as those focusing on CO, SV, and oxygen delivery optimisation. These protocols utilise advanced monitoring techniques, including arterial waveform analysis, pulse contour analysis, and echocardiography, to guide the administration of fluids and vasopressors with the aim of achieving specific haemodynamic targets. Alternatively, some protocols utilise dynamic variables like stroke volume variation (SVV) or pulse pressure variation (PPV) to guide fluid management decisions. The implementation of GDT has demonstrated notable improvements in outcomes across a range of surgical procedures, encompassing high-risk surgeries and major abdominal surgeries. The updated guidelines from the European Society of Cardiology (ESC) on cardiovascular assessment and management of patients undergoing non-cardiac surgery have assigned a Class IA recommendation for perioperative goal-directed haemodynamic therapy in high-risk surgical adult patients. Additionally, there is a strong recommendation (IB) to prevent an intraoperative decrease in MAP exceeding 20% from baseline or falling below the range of 60–70 mmHg to mitigate the occurrence of perioperative complications [23].

The use of GDT protocols has been extensively studied and implemented in the adult population; however, there is a scarcity of literature regarding their application in paediatric patients. A study conducted by Pereira de Souza Neto et al. focused on mechanically ventilated children under general anaesthesia, investigating the predictive value of dynamic parameters and transthoracic echocardiography in assessing fluid responsiveness [14]. The study findings indicated that while the respiratory variation of aortic peak velocity (ΔVpeak) proved to be an accurate predictor of fluid responsiveness, no arterial pressure or plethysmographically derived variable demonstrated accuracy in predicting fluid responsiveness.

On the other hand, Koraki et al. conducted a single-centre retrospective analysis utilising a GDT protocol based on SVV and ClearSight technology (Edwards Lifesciences) in scoliosis surgery [13]. The authors observed that this SVV and ClearSight-based GDT protocol effectively maintained haemodynamic stability and yielded favorable outcomes in patients undergoing scoliosis surgery. Notably, the protocol was associated with reduced requirements for blood transfusions, shorter hospital stays, and lower rates of postoperative complications. The results of these studies indicate the potential benefits of implementing GDT protocols in paediatric populations undergoing high-risk surgery, such as scoliosis correction. However, further research is necessary to determine the optimal approach to GDT in children and identify the most suitable protocols to improve outcomes within this specific population.

The choice of utilising an arterial waveform analysis GDT protocol, as opposed to pulse contour analysis or ultrasound analysis, is guided by multiple considerations. Firstly, arterial waveform analysis is a well-established and validated method that effectively evaluates fluid responsiveness, providing continuous and real-time feedback regarding haemodynamic fluctuations. In contrast, echocardiographic analysis necessitates specialised training and may not be feasible in all clinical settings. Furthermore, it is typically performed at specific time points and may not promptly detect acute changes in haemodynamic status. Non-invasive pulse contour analysis, on the other hand, may encounter challenges affected by factors such as hypothermia, vasoconstriction, or centralisation of circulation, ultimately compromising the accuracy of the technique.

In light of these factors, the authors have chosen to implement a GDT protocol based on SV. Alternative SVV-based protocols can pose challenges in clinical scenarios where there are variations in intra-thoracic pressure, such as during scoliosis correction. This is because SVV can be influenced by alterations in venous return and compliance, potentially leading to misinterpretation and inappropriate decisions regarding fluid management. Another aspect considered is that protocols relying on CO are more susceptible to variability due to fluctuations in heart rate, which can result in imprecise measurements, thus rendering them less dependable.

It is of utmost importance to acknowledge that during scoliosis correction procedures, the anaesthesiologist should exercise caution when implementing the conventional GDT protocol and remain mindful of its limitations in this specific surgical context. This is primarily due to instances where the surgeon exerts significant force, resulting in thoracic compression and potential kinking of the arterial cannula. Consequently, the arterial pressure waveform may exhibit a straight line pattern, which can be misinterpreted as hypotension. This ambiguity arises as the usual response to hypotension would involve initiating the GDT protocol. However, in the case of scoliosis correction procedures, it is crucial to recognise that the appropriate course of action is to adjust the force exerted by the surgeon on the surgical instruments. By appropriately modifying the applied force, it becomes possible to prevent hypotension and maintain optimal haemodynamic stability in the patient. Without clinician awareness of the importance of hypotension prevention and immediate treatment, the protocol will fail. Education and confidence in new tools is a very important part of success in hypotension prevention.

This study, despite its promising findings, is subject to several limitations that need to be acknowledged. Firstly, the study design employed was a non-randomised, single-centre, prospective, controlled cohort study, potentially restricting the generalisability of the results. The absence of randomization and the inclusion of patients solely from a single centre introduce the possibility of selection bias, thus not accurately representing the broader population of patients undergoing posterior fusion for adolescent idiopathic scoliosis. Additionally, the lack of blinding in the intervention group introduces the potential for performance bias since clinicians were aware of the treatment being administered.

Notwithstanding these limitations, this study offers valuable information regarding the potential advantages of intraoperative haemodynamic monitoring and GDT in patients with AIS. However, further exploration through larger, multicentre, randomised controlled trials is warranted to gain deeper insights and establish a more comprehensive understanding of the subject matter.

In summary, fluid management and protocolised haemodynamic monitoring have become integral elements of the paediatric ERAS (Enhanced Recovery After Surgery) protocol, offering significant potential benefits. When combined with intraoperative neuromonitoring, this approach holds promise in reducing hospital stays and minimising postoperative complications such as blood loss, neurological injuries, and wound infections. Recent studies have demonstrated that the implementation of haemodynamic optimisation and ERAS protocols can lead to improved outcomes, particularly in high-risk surgical procedures like scoliosis surgery. By employing real-time haemodynamic monitoring and goal-directed therapy protocols, healthcare providers can deliver personalised care tailored to individual patients’ physiological requirements.

As ongoing research in this field progresses, it remains crucial to continually evaluate and compare different haemodynamic monitoring and fluid management protocols. This evaluation aims to identify the most effective approaches for enhancing outcomes and minimising complications in paediatric surgical patients. Further studies are warranted to refine protocols and establish best practice guidelines specifically for scoliosis surgery.

## 5. Conclusions

To summarise, the utilisation of intraoperative haemodynamic monitoring and goal-directed therapy in patients undergoing posterior fusion for AIS has demonstrated several positive outcomes. These include reduced hospital stay duration, shorter intraoperative hypotension time, and improved preservation of haemoglobin and haematocrit levels.

## Figures and Tables

**Table 1:** Demographic data from patients among the groups.

Parameters	Control (*n* = 35)	Intervention (*n* = 35)	*p*
Age	15 (14–17) *	14 (13–17) *	0.144
Gender (boys/girls)	7/28	5/30	0.529
BMI	20.58 (18.43–22.36) *	18.97 (17.93–21.77) *	0.259
Haemoglobin level before surgery (g/dL)	13.71 (1.326) **	13.03 (0.93) *	0.016
Haematocrit level before surgery (%)	38.29 (4.03) **	37.93 (2.72) **	0.672
ASA * 1	21	22	0.297
ASA 2	14	10	
ASA >2	0	3	

Legend: BMI—body mass index, ASA—American Society of Anaesthesiology; * median, interquartile range; ** mean, standard deviation.

**Table 2:** Perioperative data, time of hypotension, complications, transfusions.

Parameters	Control (*n* = 35)	Intervention (*n* = 35)	*p*	FDR	Power
Duration of the surgical procedure “skin to skin” (minutes)	195 (16–218) *	205 (170–240) *	0.390	0.442	0.52
Neurological complications (no)	1	0	0.5	0.531	0.32
Cardiac complications (no)	3	0	0.239	0.290	0.66
Duration of hospitalisation (days)	10 (9–11) *	7 (6–8) *	<0.001	0.003	0.99
Hypotension time (min)	40 (20–60) *	8 (1–14) *	<0.001	0.003	0.98
Red blood cells transfusion volume (mL)	290 (0–560) *	190 (0–290) *	0.123	0.16	0.48
Fresh frozen plasma transfusion volume (mL)	200 (0–200) *	0 *	<0.001	0.003	0.99
Intraoperative blood loss (mL)	500 (350–500) *	500 (350–588) *	0.903	0.903	0.07
Crystalloids administered (mL/kg)	22 (16.3–31.5) *	27.8 (20.1–35.4) *	0.078	0.121	0.4
Ephedrine total dose (mg)	0 (0–8.75) *	5 (0–13.75) *	0.098	0.139	0.05
Intraoperative fluid administration (mL)	23.5 (10.05)	27.8 (9.93)	0.078	0.121	0.4
Haemoglobin level after surgery (g/dL)	9.71 (1.59) **	10.67 (1.02) **	0.007	0.017	0.74

Change in haemoglobin ^ (g/dL)	−3.83 (1.50) **	−2.36 (1.14) **	<0.001	0.006	0.97
Haematocrit level after surgery (%)	27.93 (4.05) **	31.05 (3.18) **	0.002	0.064	0.87
Change in haematocrit level ^ (%)	−9.53 (5.39) **	−6.89 (3.69) **	0.030	0.003	0.53
Time to extubation ^$^ (min)	27.5 (20–50) *	0 (0–15) *	<0.001	0.115	0.99
Intraoperative diuresis (mL)	135 (100–250)	215 (127–300)	0.061	0.442	0.21

Legend: * median, interquartile range; ** mean, SD; ^ before-after surgery; ^$^ time from end of operation till extubation.

## Data Availability

The raw data are available upon request from the corresponding author.
